# Management of Complications during Gastric Endoscopic Submucosal Dissection

**DOI:** 10.1155/2012/624835

**Published:** 2012-10-03

**Authors:** Dong Wook Lee, Seong Woo Jeon

**Affiliations:** ^1^Internal Medicine, Daegu Fatima Hospital 183, Ayang-ro, Dong-gu, Daegu, Republic of Korea; ^2^Internal Medicine, Kyungpook National University School of Medicine, 50 Samduk 2Ga, Chung-gu, Daegu, Republic of Korea

## Abstract

Popularity of endoscopic submucosal dissection (ESD) has shown an increase during the last decade, and may, for the time being, be the most important technique in treatment of early gastrointestinal cancer or a premalignant lesion. This technique has advantages in the aspect of en bloc resection, which enables evaluation of the completeness of resection and other pathologic characteristics; however, it has limitation in terms of complications, compared to endoscopic mucosal resection (EMR). Bleeding and perforation are the most common complications encountered during the procedure. These complications can cause embarrassment for the endoscopist and hamper performance of the procedure, which can result in an incomplete resection. To overcome these obstacles during performance of the procedure, we should be familiar with management of complications. In particular, beginners who start performing ESD should have full knowledge of and be in good handling of the method of hemostasis using hemoclips or electrocoagulation for management of complications. Various methods, procedures, and equipment are under development, which will provide us with powerful tools for achievement of successful ESD without complications in the near future.

## 1. Introduction

ESD (endoscopic submucosal dissection) is widely used as a treatment for early cancerous and premalignant lesions in the gastrointestinal tract. When compared with the conventional EMR (endoscopic mucosal resection) method, ESD presents various advantages, such as a higher degree of complete resection and en bloc resection; however, it is also associated with a higher potential for complications, such as perforation or bleeding [1–3]. This chapter examines bleeding and perforation, which are complications that can occur during ESD, and investigates measures for dealing with such complications.

## 2. Management of Bleeding

Immediate bleeding was defined as bleeding that occurred during the ESD procedure. The degree of bleeding has not been validated yet; however, a recent study presented the grading system as follows: grade 0 (no visible bleeding during procedure), grade 1 (trivial bleeding which stops spontaneously or easily controlled by single session of hemocoagulation), grade 2 (minor bleeding which controlled by multiple sessions of hemocoagulation or easily controlled by hemoclips), and grade 3 (major bleeding which needs multiple hemoclips and hemocoagulation) [4]. Delayed bleeding was generally defined as one of the following: hematemesis or melena, unstable vital signs or hemoglobulin loss >2 g/dl after the ESD procedure [4, 5].

### 2.1. Management of Immediate Bleeding

Bleeding generated during ESD can be classified according to immediate and delayed bleeding. Immediate bleeding commonly develops in the body rather than in the antrum of the stomach. The body is composed of many large blood vessels; therefore, operators must perform ESD with special caution. Thus, sufficient submucosal injection and performance of precutting at a moderate depth while closely observing blood vessels are important. 

**Figure 1:**
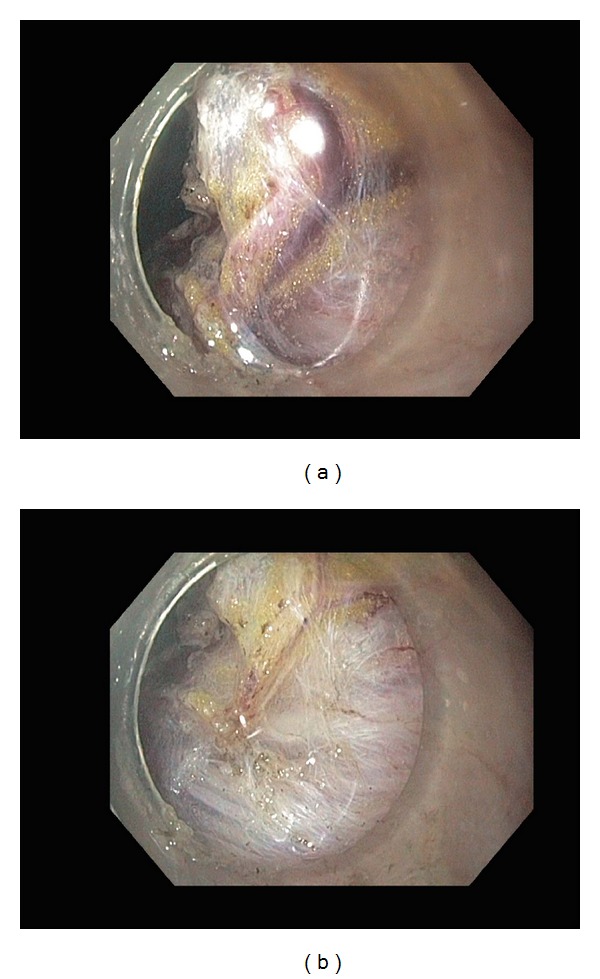
(a) A large vessel was noted during submucosal dissection. (b) After preventive coagulation using hemostatic forceps, a whitish stigma was observed without evidence of bleeding.

If large blood vessels are observed during dissection, in order to decrease immediate bleeding, it is more effective to continue dissection after performing hemostasis through use of hemostatic forceps or coagulation probes (Figure 1). To achieve this, attachment of a transparent cap to the tip of the endoscope during ESD for performance of dissection with direct vision of the submucosal layer of the lesion can be very helpful. According to a study investigating predictive factors of immediate bleeding, rate of occurrence of bleeding was 12%, and the possibility of bleeding was lower in older patients with lesions located in the antrum [4]. In addition, the possibility of bleeding did not show an association with sex or the size/shape of the lesion. Other studies have reported generation of bleeding in 7% of patients, which was a common occurrence if the lesion was located in the upper or mid portion of the stomach and if it was larger than 3 cm. ESD should be performed with caution, as bleeding disrupts the operator's sight, which may result in an increase in procedure time and the potential for other complications. Also, prompt hemostasis must be performed if immediate bleeding develops. Among accessories released to date, there have been no effective tools for achievement of both dissection and hemostasis. If immediate bleeding develops in small vessels, a dissection knife can be used in performance of hemostasis through coagulation mode (swift or forced coagulation for VIO 300D, ERBE, Germany). However, this may be insufficient for large blood vessels and may rather cause formation of carbonization, resulting in aggravated bleeding and disruption of the operator's sight. In this situation, it is more effective to select an appropriate tool, such as a hemostatic forceps or coagulation probe, according to the preference of the operator, for performance of hemostasis through coagulation mode (soft coagulation for VIO 300D) or argon plasma coagulation. Although it is preferable to avoid use of the hemoclip, as it may interfere with performance of the procedure, if it must be used for inevitable reasons, the operator must take care to prevent it from interfering with the procedure after hemostasis, such as dissection.

### 2.2. Management of Delayed Bleeding

Several studies have reported delayed bleeding at various rates, ranging from 1.8% to 15.6% [4, 5]. These wide ranges of incidence are resulting from the differences in the definition of delayed bleeding and retrospective design. In a study analyzing the results of ESD in 1000 cases, risk factors for delayed bleeding included location (upper portion), size (≥4 cm), shape (flat) of the lesion, and recurrence of lesions [5]. In another study of the predictive factors of delayed bleeding, occurrence was reported in 5.8% of lesions and 6.5% of patients. Only one patient required blood transfusion. Bleeding was significantly lower if the tumor location was upper third or post-ESD coagulation was performed [6]. This is not only because lesions are more common in the antrum, but is also associated with the following reasons: the operator can care less about bleeding as ESD can be easily performed in antrum lesions, antrum lesions have greater exposure to bile juice, when compared with the upper portion, and potential for bleeding is greater with antrum lesions due to physical friction resulting from active stomach movements. Methods for completely stopping delayed bleeding have yet to be developed, and complete blockage cannot be achieved even if a second look endoscopy is performed on the day after ESD [7]. Thus, achievement of complete hemostasis of blood vessels that present the possibility of bleeding during ESD is important; furthermore, delayed bleeding can be reduced through prophylactic hemostasis [8]. 

## 3. Management of Perforation

Although occurrence of perforation is rather uncommon during ESD, when compared with bleeding, it is a complication that is experienced by operators at one time or another. As perforation can occur during ESD, even if the operator takes special precautions, it is more appropriate to express perforation as an epiphenomenon rather than a complication [9]. Thus, ESD operators must be equipped with the ability to deal rapidly with perforations. 

**Figure 2:**
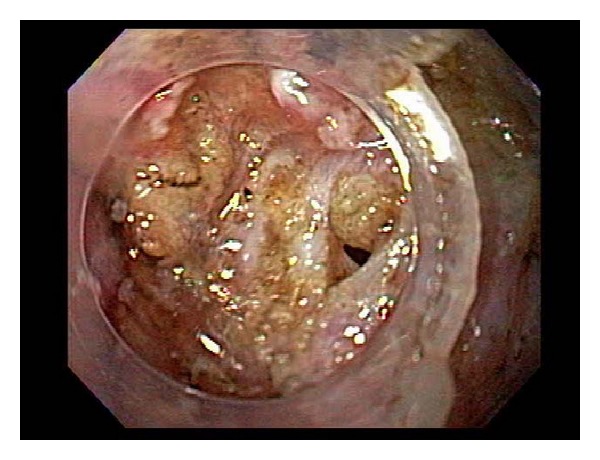
Example of macroperforation during submucosal dissection. The perforation was closed with hemoclips and the tumor was resected en bloc with snare.

Perforation during ESD can be classified according to microperforations, which are not visible, and large macroperforations, which are visible (Figure 2). Perforations can also be classified according to the period of occurrence, thus, perforation generated during ESD and delayed perforation. Macroperforation can be diagnosed when the patient complains of abdominal pain during ESD, when the stomach is not distended during air inflation, when abdominal distension is checked, and when the area of perforation is checked through an endoscope. Because observance of free air on the chest X-ray taken after ESD generally leads to a diagnosis of microperforation, conduct of radiologic examinations after ESD is important. 

Perforations generally present in the proximal body rather than the antrum of the stomach [10]. This is because, although fewer perforations are generated in the antrum, as ESD can be easily performed in this area based on favorable accessibility of the endoscope, gaining access to the body is difficult, and the gastric wall is thin. In particular, the risk of perforation is high in relation to few internal oblique muscles and the difficulty of producing cushion in lesions located in the greater curvature or in the anterior wall of the distal body during administration of submucosal injection. 

Perforations during ESD occur mainly during precutting or dissection of lesions and rarely occur as a result of defective handling of the endoscope. Perforations are occasionally generated during hemostasis. According to one study, the frequency of perforation was measured as 1.2% [5]. In a study establishing the clinical results of perforation for a total of 1,711 patients, development of perforation was reported in 39 cases (2.3%), whereas macroperforation accounted for 67% of total cases of perforation. Occurrence of perforation was more common during submucosal dissection than during precutting. Only one patient underwent surgery due to severe bleeding and perforation, while others showed improvement as a result of endoscopic treatment or conservative treatment. No differences in clinical outcome were observed between microperforation and macroperforation. In a study comparing occurrence of perforation according to the experience of the operators by year, it was determined that the rate of occurrence did not differ significantly according to the experience of the operators. In this regard, it is more appropriate to view perforation as an epiphenomenon than a complication [11].

When perforation occurs, achievement of effective closure is important, in order to prevent surgery. In most cases, if successful closure is achieved, the patient can be treated through endoscopic management. This is because NPO conducted before ESD decreases the content that can be contaminated within the stomach and reduces the potential for development of bacteremia due to stomach acid. If perforation occurs, a clear sight must first be obtained while minimizing air inflation. Irrigation of the lesion with water in order to obtain a clear sight can cause peritonitis. Once the lesion has been completely closed using hemoclips, performance of ESD can continue. Operators must remember that the hemoclip can interfere with the procedure, resulting in incomplete resection. In addition, if the patient is in an unstable condition due to severe peritoneal irritation, ESD must be continued by removal of air through paracentesis and should be completed as rapidly as possible. After ESD, patients must be observed closely with NPO and administration of antibiotics. If the patient's condition is aggravated with fever and persistent symptoms of peritoneal irritation, surgery must be considered. If symptoms are not aggravated, the patient must be observed closely for a period of 2–4 days in order to determine whether the patient can stop fasting and be discharged from the hospital. 

The reason for formation of intraperitoneal free air during microperforation is unclear; however, air leakage may develop due to formation of invisible perforations or due to increased stomach pressure caused by excessive air inflation resulting from formation of burns in the muscle layer and thinning of the remaining wall generated by coagulation. Operators do not have to eliminate intraperitoneal free air in all cases. However, removal of free air aids in relief of symptoms if the patient has severe abdominal pain and symptoms of peritoneal irritation [12]. 

If perforation occurs, operators can insert an nasogastric tube in order to eliminate gastric contents, and to block dissemination into the peritoneal cavity [13, 14]. However, certain studies have reported that this is not absolutely necessary; therefore, doctors must practice appropriate management of such situations, based on experience, and additional evidence must be gathered in relation to this issue [11]. 

Although delayed perforation is not common, it can occur in certain patients. Surgical treatment is required in most cases. The prophylactic measure to reduce or eliminate the delayed perforation has not been studied. However, delayed perforation could be prevented with skillful procedures. Avoiding muscular injury during procedure is essential. The muscular injury could be the initial insult of microperforation, and it could lead to frank perforation without recognition. This disastrous event also could be kept away if routine radiographic study after ESD was available. Some endoscopists insist that this strategy is not effective in patients without symptoms of perforation. However, I assume that almost all delayed perforation develop in overlooked cases, which already had severe gastric muscular injury or microperforation immediately after ESD. It might be difficult to prove this hypothesis due to low incidence of delayed perforation; however, clinical practice of routine radiologic examination after ESD should be kept in mind. 

## 4. Management of Other Complications

**Figure 3:**
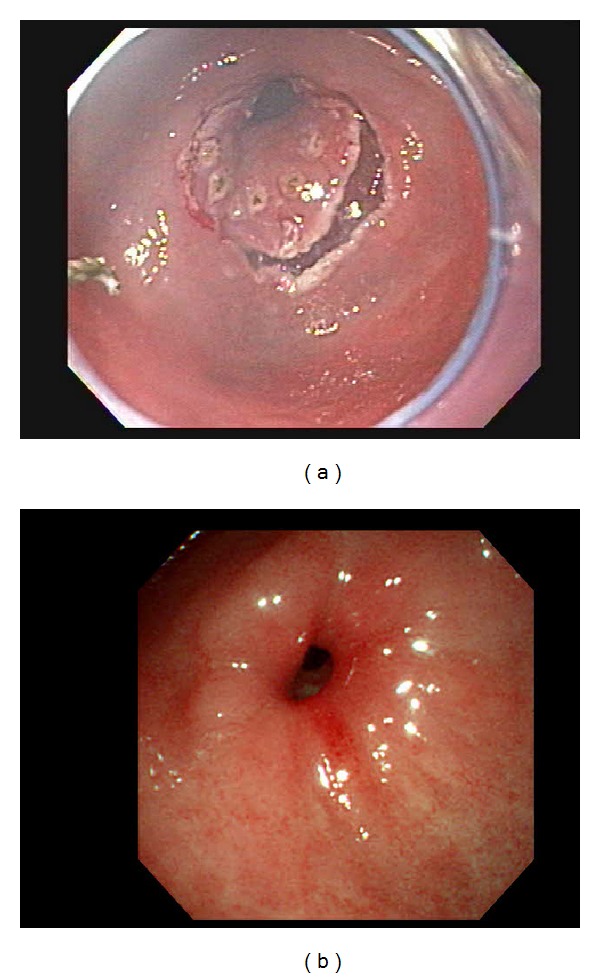
(a) A large nodular lesion involving pylorus was observed at the antrum. (b) Follow-up endoscopy showed luminal stenosis, but without obstructive symptoms.

In addition to bleeding and perforation, stenoses of cardia/pylorus and aspiration pneumonia are also frequently observed complications. In a recent analysis of 2011 cases of EGC, development of stenosis was reported in 17% of cardia and 7% of pylorus when ESD was performed for lesions located within 1cm of the EG junction or pylorus. Both areas showed an association with stenosis if the lumen was dissected by more than 3/4 or if the long axis of resection length was more than 5 cm. Balloon dilatation was performed successfully in all cases [15]. Treatment can be achieved through balloon dilatation, even in cases involving formation of stenosis. However, in order to prevent any problems, it is important to explain the possibility of complications to patients (Figure 3). For older patients, aspiration pneumonia can develop after ESD. In particular, because the patient takes the left decubitus position during ESD, formation of aspiration pneumonia often occurs in the left side lung. However, improvement of aspiration pneumonia can be achieved through antibiotic treatment. Operators must perform frequent suction of gastric fluid during ESD. Avoidance of excessive air expansion helps to prevent development of aspiration pneumonia.

## 5. Conclusion

ESD is a procedure that has been established as a significant part of treatment for EGC and premalignant lesions. For effective performance of ESD, it is important to take appropriate measures for management of complications and to be equipped with the basic knowledge and skills. In particular, for appropriate management of complications, beginners who are performing ESD for the first time must have full knowledge of the performance of hemostasis using hemoclips or electrocoagulation.

In the future, various methods, procedures, and equipment must be developed in order to create better ways of handling complications that occur during ESD. 

## Abbreviations

ESD: Endoscopic submucosal dissection
EMR: Endoscopic mucosal resection
EGC: Early gastric cancer.
